# Baseline Values and Kinetics of IL-6, Procalcitonin, and TNF-*α* in Landrace-Large White Swine Anesthetized with Propofol-Based Total Intravenous Anesthesia

**DOI:** 10.1155/2021/6672573

**Published:** 2021-06-19

**Authors:** Athanasios Chalkias, Vaios Spyropoulos, Georgia Georgiou, Eleni Laou, Anastasios Koutsovasilis, Ioannis Pantazopoulos, Konstantina Kolonia, Spyros Vrakas, Apostolos Papalois, Styliani Demeridou, Konstantinos Gourgoulianis, Ismene Dontas, George Kaparos, Stavroula Baka, Theodoros Xanthos

**Affiliations:** ^1^Department of Anesthesiology, University of Thessaly, School of Health Sciences, Faculty of Medicine, Larisa, Greece; ^2^Outcomes Research Consortium, Cleveland, OH 44195, USA; ^3^Medical Supervision S.A. Private Medical Center, Athens, Greece; ^4^Department of Surgery, National and Kapodistrian University of Athens, 1st Propaedeutic Surgical Clinic, Athens, Greece; ^5^Department of Internal Medicine, Nikaia General Hospital, Nikaia, Greece; ^6^Department of Emergency Medicine, University of Thessaly, School of Health Sciences, Faculty of Medicine, Larisa, Greece; ^7^Department of Gastroenterology, Tzaneio General Hospital, Piraeus, Greece; ^8^Experimental-Research Center “ELPEN” Pharmaceutical Co, Athens, Greece; ^9^Department of Biopathology-Microbiology and Biochemistry, National and Kapodistrian University of Athens, Aretaieion University Hospital, Athens, Greece; ^10^Department of Respiratory Medicine, University of Thessaly, School of Health Sciences, Faculty of Medicine, Larisa, Greece; ^11^National and Kapodistrian University of Athens, Medical School, Laboratory for Research of the Musculoskeletal System, KAT General Hospital, Athens, Greece; ^12^European University Cyprus, School of Medicine, Nicosia, Cyprus

## Abstract

The baseline levels of various inflammatory mediators and their changes during anesthesia in swine are not known. The aim of this animal study was to measure the baseline values and kinetics of interleukin-6, procalcitonin, and tumor necrosis factor-alpha in healthy Landrace-Large White swine anesthetized with propofol-based total intravenous anesthesia. We included 8 healthy male pigs with an average weight of 19 ± 2 kg (aged 10-15 weeks) that were subjected to propofol-based total intravenous anesthesia for 8 hours. Complete blood count, serum chemistry, and serum levels of interleukin-6, procalcitonin, and tumor necrosis factor-alpha were analyzed, and serum levels were quantified hourly. Blood was also collected for bacterial culturing. Baseline values of interleukin-6 and procalcitonin were 18 pg/ml and 21 ng/ml, respectively, while tumor necrosis factor-alpha was not detectable during collection of baseline samples. A statistically significant difference was observed in interleukin-6 levels between time points (*p* < 0.0001). Procalcitonin increased with time, but there were no significant differences between time points (*p* = 0.152). Tumor necrosis factor-alpha increased until the 3rd hour of propofol-based total intravenous anesthesia, while after the 4th hour, it gradually decreased, reaching its baseline undetectable values by the 7th hour (*p* < 0.001). Our results can serve as the basis for further translational research.

## 1. Introduction

Systemic inflammatory responses are pivotal in the pathogenesis and progression of several acute and chronic diseases. These responses can be induced by several procedures and are an inherent response of the body to various stressful events. Notably, immune system dysfunction has been suspected to influence disease progression, morbidity, and mortality, especially in critically ill patients.

Anesthetics are currently used in various settings, mostly in the operating room and the intensive care unit (ICU). Many *in vivo* studies have suggested that the immunomodulatory effects of anesthesia are negligible in comparison to the processes triggered by tissue injuries [1]. However, the effect of anesthetics on the immune system has been reported from modest and without clinically significant effects to severe with devastating consequences [2]. Anesthesia-induced immune responses may affect the expression of proinflammatory cytokines and may vary depending on the intensity of other interventions or conditions, e.g., trauma [3]. Although cytokines are major modulators of inflammation, cytokine dysregulation may lead to immunosuppression and multiple organ dysfunction or infectious disorders [4], which may be extremely important in case of states or diseases characterized by a cytokine increase.

Research on anesthesia-related immunomodulation increased during the last decades, but the clinical implications of these findings have not been satisfactorily defined. The effect of anesthetics on immune system remains a matter for controversial discussion, mainly because baseline (normal) levels of various inflammatory mediators and their change during maintenance of anesthesia have not been adequately described in all species. Moreover, anesthetics have not been extensively studied isolated without any concomitant surgical intervention [1]. Another main problem of anesthesia worldwide is cognitive disorders, either postoperative or after ICU admission and discharge. Knowing the effects of anesthetics *per se* will help us to optimize personalized physiology-guided treatment and enhanced recovery protocols. Moreover, this knowledge may be used in translational research, enhancing the translation of future findings from bench to the bedside and *vice versa*.

In this investigation, the aim was to measure the baseline values of interleukin-6 (IL-6), procalcitonin (PCT), and tumor necrosis factor-alpha (TNF-*α*) in healthy Landrace-Large White swine anesthetized with propofol-based total intravenous anesthesia (PTIVA), as well as to study their kinetics during maintenance of anesthesia.

## 2. Materials and Methods

### 2.1. Ethical Approval

In order to follow the guiding principles underpinning the human use of animals in scientific research (three Rs) [5, 6], the animals included in the present study were recruited from the control group of another study of our research group (Licence no. 26/10-01-2012) [7]. The original study protocol was approved by the Greek General Directorate of Veterinary Services and was conducted in accordance to the Greek legislation regarding ethical and experimental procedures (Presidential Decree 160/1991, in compliance with the EEC Directive 86/609 and Law 2015/1992 and in conformance with the European Convention for the protection of vertebrate animals used for experimental or other scientific purposes, 123/1986).

### 2.2. Origin and Source of the Animals

Study subjects were 8 healthy male Landrace-Large White pigs, aged 10-15 weeks, with an average weight 19 ± 2 kg, and of conventional microbiologic status. Male animals were used for homogeneity and in order to minimize the possible effect of sex hormones. The animals were purchased from the same breeder (Validakis, Koropi, Greece). All animals were housed in single cages. The conditions in the animal house were 15 air changes hourly, 22 ± 2°C, 55% relative humidity, lights on at 06 : 00 am, and lights off at 06 : 00 pm. The animals were transported one week before experimentation to the research facility (Experimental Research Center, ELPEN, European Ref No. EL 09 BIO 03) to acclimatized to laboratory conditions and were fed a standard commercial food (Biozokat, Ekaterinis-Larissis, Greece) [8, 9]. The day before the experimentation, the animals were fasted but had free access to water. All animals received anesthetic and surgical procedures in compliance with the Guide for the Care and Use of Laboratory Animals.

### 2.3. Animal Preparation

Animals were premedicated with intramuscular injection of 10 mg/kg ketamine hydrochloride (Merial, Lyon, France), 0.5 mg/kg midazolam (Roche, Athens, Greece), and 0.05 mg/kg atropine sulphate (Demo, Athens, Greece), as previously described [8]. The animals were subsequently transported to the operation research facility, and intravascular access through the auricular veins was obtained. Anesthesia was induced by an intravenous bolus dose of 2 mg/kg propofol (Diprivan 1% wt/vol; Astra Zeneca, Luton, United Kingdom) and 2 *μ*g/kg fentanyl (Janssen Pharmaceutica, Beerse, Belgium) [9, 10]. The same researcher performed the intubation while the animals were breathing spontaneously with a 5.0 mm endotracheal tube (Portex, 5.0 mm ID; Mallinckrodt Medical, Athlone, Ireland). The endotracheal tube was secured on the lower jaw, and successful intubation was ascertained by auscultation of both lungs while ventilated with a self-inflating bag [8–10]. The animals were then immobilized in the supine position on the operating table, and additional 1 mg/kg propofol, 0.15 mg/kg cis-atracurium (Nimbex 2 mg/ml; GlaxoSmithKline, Athens, Greece), and 0.01 mg/kg fentanyl were administered to achieve synchrony with the ventilator.

The animals were volume-controlled ventilated (tidal volume 10 ml/kg, I : E 1 : 2, PEEP 0 cm H_2_0, and FiO_2_ 21% (Siare Alpha-Delta Lung Ventilator; Siare s.r.l. Hospital Supplies, Bologna, Italy)) [10]. Propofol infusion of 100 *μ*g/kg/min, cis-atracurium at 20 *μ*g/kg/min, and fentanyl at 0.6 *μ*g/kg/min were administered to maintain adequate anesthetic depth throughout the study [8, 10]. No extra bolus doses were administered, while the infusion rates were maintained constant throughout the study. We used the assessment of jaw tone throughout the experiment to assess the anesthetic depth according to the guidelines on anesthesia and analgesia in Swine [9]. Normocapnia was achieved using continuous monitoring of end-tidal CO_2_ (Tonocap TC-200-22-01; Engstrom Division, Instrumentarium Corp, Helsinki, Finland), and the respiratory rate was adjusted to maintain end-tidal CO_2_ 35-40 mmHg. Pulse oximetry (SpO_2_) was monitored throughout the experiment. Body temperature was monitored by a rectal temperature probe and was maintained between 38.5°C and 39.5°C with a heating blanket [9].

Electrocardiographic monitoring was used using leads I, II, III, aVR, aVL, and aVF, which were connected to a monitor (Mennen Medical, Envoy; Papapostolou, Athens, Greece). The monitor electronically calculated the heart rate. For measurement of the aortic pressures, an arterial catheter (model 6523, USCI CR, Bart; Papapostolou) was placed in the right femoral artery [8, 10]. The systolic (SAP) and diastolic (DAP) arterial pressures were recorded, whereas mean arterial pressure (MAP) was determined by the electronic integration of the aortic blood pressure waveform [11]. A catheter (18 gauge; Vitroflon, Vitromed Healthcare, India) was also placed into the left femoral vein. Prior to each catheter placement, the skin was prepared with 2% chlorhexidine gluconate/70% isopropyl alcohol. Arterial blood gases were measured on a blood-gas analyzer (IRMA SL Blood Analysis System, Part 436301; Diametrics Medical Inc, Roseville, MN 55113).

The animals were monitored for 8 hours without any further intervention except blood sampling in order to eliminate the effects of other causes on immune response [12]. Subsequently, anesthesia was discontinued, all catheters were removed as previously described, and manual ventilation was initiated [8–10]. Atropine 0.2 mg/kg followed by neostigmine 0.05 mg/kg was administered when spontaneous swallowing reflex was detected, whereas extubation was performed after adequate inspiration depth was confirmed [8, 10]. Each animal was then transferred to the animal house.

### 2.4. Blood Sampling and Analysis

During the 8-hour experimental procedure, 20 ml of blood was collected after instrumentation (baseline) and then hourly from the left femoral vein of each animal. Complete blood count was analyzed in 5 ml ethylenediaminetetraacetic acid tubes on an automated hematology analyzer XS-1000 (Sysmex, Kobe, Japan). Serum samples were prepared by centrifugation of whole blood at 3300 rpm for 10 min at 8°C. Complete blood count included white blood cells, platelets, neutrophils, lymphocytes, hemoglobin, and hematocrit. Serum chemistry analysis included alanine aminotransferase, aspartate aminotransferase, gamma-glutamyl transferase, creatinine, urea, creatine kinase, and C-reactive protein, measured on a Konelab 30i chemistry analyzer (Thermo Scientific, Massachusetts, United States). Serum levels of IL-6, PCT, and TNF-*α* were quantified by corresponding pig sandwich enzyme-linked immunosorbent assays (ELISA) (Cusabio Technology LLC, Houston, United States), with the assay sensitivity being 0.03 pg/ml, 7.81 ng/ml, and 11.7 pg/ml, respectively. Intra-assay and interassay precisions were less than 8% and 10%, respectively, for all assays.

### 2.5. Blood Cultures

Blood was collected via the peripheral venous catheter for bacterial culturing at baseline, 4 hours and 8 hours. Prior to each blood drawing, the catheter was prepared with 70% isopropyl alcohol. Blood was inoculated into a BD Plus Aerobic/F Culture Vial (8-10 ml), a BD Plus Anaerobic Blood Culture Vial (8-10 ml), and a BD Mycosis Culture Vial (8-10 ml). Blood culture specimens were entered into an incubation protocol on a BD BACTEC 9050 Blood Culture System (BD India Pvt. Ltd, Haryana, India) and were continuously monitored for five days or until the system detected a positive culture.

### 2.6. Statistical Analysis

Continuous variables were expressed as mean ± SD and categorical variables as percentages. The normal distribution of each variable was tested by Kolmogorov-Smirnov's test. Comparisons of continuous variables among the groups were made using analysis of variance (ANOVA) or the Kruskal-Wallis test, as appropriate. Pearson correlation was used to explore possible correlations between the hemodynamics variables and the proinflammatory cytokines. All tests were two tailed, and a probability value of *p* < 0.05 was considered significant. All statistical analysis was performed using SPSS version 21.0 (SPSS Inc, Chicago IL, USA).

## 3. Results and Discussion

### 3.1. Results

**Figure 1:**
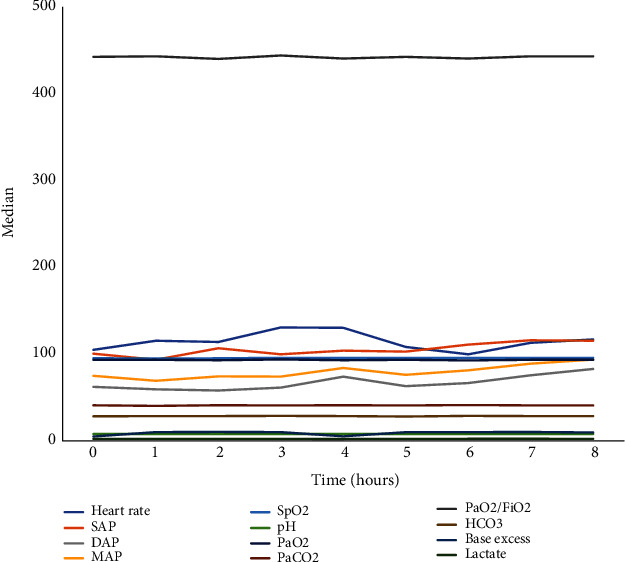
Hourly fluctuation of hemodynamic and metabolic parameters. SAP: systolic arterial pressure; DAP: diastolic arterial pressure; MAP: mean arterial pressure; SpO_2_: peripheral oxygen saturation; PaO_2_: arterial partial pressure of oxygen; PaCO_2_: arterial partial pressure of carbon dioxide; FiO_2_: fraction of inspired oxygen.

We observed a statistically significant fluctuation in heart rate (*p* = 0.041), SAP (*p* = 0.038), DAP (*p* = 0.036), and MAP (*p* = 0.044) during the 8-hour PTIVA. We did not observe statistically significant differences in metabolic parameters between time points (Table 1 and Figure 1). According to Pearson correlation coefficient, there was a correlation between heart rate and IL-6 (*r* = 0.227, *p* = 0.031), heart rate and PCT (*r* = 0.421, *p* = 0.029), DAP and IL-6 (*r* = −0.203, *p* = 0.045), DAP and PCT (*r* = −0.404, *p* = 0.036), MAP and PCT (*r* = −0.492, *p* = 0.009), and MAP and TNF-*α* (*r* = 0.510, *p* = 0.007).

A statistically significant difference during the 8-hour experimental period was observed only in urea (*p* = 0.010). We did not find significant differences in any other parameter of the complete blood count and serum chemistry analysis between time points (Table 2). Baseline IL-6 levels were 18 pg/ml and fluctuated during the 8-hour study period. We observed a statistically significant difference between the time points (*p* < 0.0001) and two peaks compared to baseline value, a higher peak at 2 hours and a lower peak at 7 hours. Baseline PCT values were 21 ng/ml. Although PCT was gradually increased during the 8-hour PTIVA compared to its baseline values, there were no significant differences between the time points of the study (*p* = 0.152).

**Figure 2:**
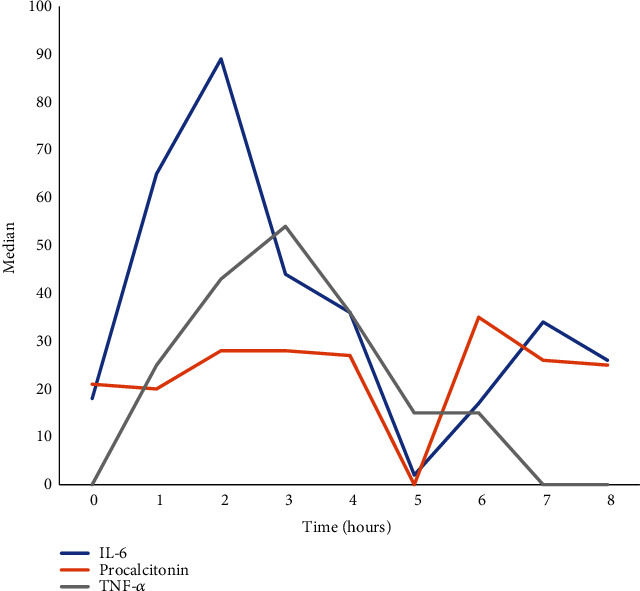
Hourly fluctuation of IL-6, PCT, and TNF-*α* during anesthesia.

Tumor necrosis factor-alpha was not detectable at baseline. However, it was gradually increased until the 3rd hour of PTIVA. After the 4th hour of PTIVA, TNF-*α* began to decrease, being undetectable again by the 7th hour of PTIVA (*p* < 0.001) (Table 3 and Figure 2). All the specimens were negative after the end of the 5-day incubation period.

## 4. Discussion

Inflammatory conditions and cytokine upregulation have not been studied sufficiently in swine due to the lack of baseline values for these mediators. In this study, we first report the baseline values and kinetics of IL-6, PCT, and TNF-*α* in healthy Landrace-Large White swine anesthetized with PTIVA. Our results open the way for further translational research in this species, which is important considering that the hemodynamics of Landrace-Large White closely resemble human hemodynamics [7–11, 13].

Propofol-based total intravenous anesthesia is a widely used method for induction and maintenance of anesthesia. Propofol may exert mixed pro- and anti-inflammatory effects on the immune system of healthy subjects [14, 15]. It inhibits cyclooxygenase-2, reducing the production of prostaglandin E2, preserves the function of natural killer cells, diminishes the production of cytokines, and enhances activation and differentiation of peripheral T-helper cells which augment cellular immunity [16]. Also, propofol has been reported to inhibit lipid peroxidation in various experimental models, to protect cells against oxidative stress, and to increase the antioxidant capacity of plasma in humans [17, 18]. Propofol may decrease the expression of NFkB, an important transcription factor with a key role in oxidative stress and inflammatory responses activated during ischemia/reperfusion, thus decreasing neutrophil infiltration, oxygen-derived free radicals, and the production of proinflammatory cytokines [19]. It can therefore be suggested that propofol's antioxidant properties might be relevant to organ protection during anesthesia. On the other hand, several studies have shown the stimulating effects of propofol on cytokine production, e.g., in critically ill patients in whom propofol may increase serum levels of IL-1*β*, IL-6, and TNF-*α* [20].

In this study, we observed a nonsignificant increase in white blood cells and neutrophils during the 8-hour period, but the negative blood cultures indicate a noninfectious inflammatory response. Our animals were premedicated with midazolam and ketamine and were anesthetized with propofol and fentanyl. Midazolam may modulate the immune system by binding to the peripheral receptor of macrophages and inhibiting the proinflammatory responses [21], while ketamine acts at different levels of inflammation, interacting with inflammatory cell recruitment, cytokine production (IL-6, TNF-*α*), and regulation of inflammatory mediators [12], which could explain in-part the nonsignificant increase in white blood cells. Also, propofol exerts inhibitory effects on neutrophil and monocyte function under different physiological and pathological circumstances, while the effects of cisatracurium and fentanyl are diverse in different disease states and must be further studied [22, 23].

Interleukin-6 is a cytokine with an extensive range of biological activities and is active during the process of inflammation [24]. We chose to evaluate IL-6 because it is the main cytokine responsible for inducing the systemic changes known as the acute-phase response. Interleukin-6 may stimulate the hypothalamic-pituitary-adrenal axis and has been reported to produce postoperative neurological dysfunction in mice [12]. In addition, IL-6 may induce neutrophil activation and delay the phagocytic disposal of senescent or dysfunctional neutrophils, thereby prolonging their injurious effects, can contribute to myocardial dysfunction and cardiomyocyte necrosis in ischemia-reperfusion, and may promote organ injury [1, 9, 13]. In our study, IL-6 fluctuated during the 8-hour period, and we observed statistically significant differences between time points. In addition, IL-6 was characterized by two peaks, a higher one at 2 hours and a lower one at 7 hours compared to its baseline level. These findings may be attributed to the effects of anesthetics and the absence of major surgical stress [1, 8, 12]. However, our study included healthy animals, and in patients with comorbidities or in critically ill patients, even modest anesthetic doses or different duration of anesthesia may result in different IL-6 fluctuations. In a previous study investigating the development of local inflammation in glottic tissues, we reported a positive correlation between IL-6 levels and duration of intubation/PTIVA [25]. A high IL-6 level has been associated with postoperative complications and lower survival rate in critically ill patients [26], while upregulation of IL-6 may affect neuronal precursor cells, resulting in long-lasting influences on neuronal development and metabolism [27].

Procalcitonin is used as a biomarker to aid in diagnosis of bacterial infection or sepsis, and its serum concentration in healthy individuals is typically <0.1 ng/ml. However, baseline PCT values had not been reported in swine until now. In our study, baseline PCT was 21 ng/ml and increased during the 8-hour PTIVA. Although we did not observe statistically significant differences between measurements, PCT fluctuated at significantly higher values than the normal ones in humans. In general, PCT levels > 10 ng/ml are almost exclusively due to severe bacterial sepsis or septic shock, and therefore, the interpretation of PCT in our study is challenging. The mechanism by which general anesthesia may increase the incidence of infection is not fully understood, but accumulating evidence suggests a role for immunosuppression [28]. On the contrary, the negative results of blood cultures and the kinetics of PCT indicate a noninfectious inflammatory stimulus [29]. Interestingly, PCT may act as a chemokine, influencing the migration of monocytes and parenchymal cells. Dysregulated PCT production leading to high PCT levels may also be seen in paraneoplastic syndromes and carcinomas [30], but we did not find evidence of such diseases in our swine. It remains unknown whether an anesthesia-induced sterile inflammatory response increases PCT levels, and it is recommended that further research be undertaken in this area.

Tumor necrosis factor alpha is a proinflammatory cytokine participating in many physiological and pathophysiological processes. It is a powerful inducer of inflammation, and it may promote monocyte/macrophage differentiation and tumor cell necrosis/apoptosis [31]. However, overproduction of TNF-*α* can be disastrous to the host by stimulating immune cell infiltration, cell death, and eventually organ damage [27]. In our animals, TNF-*α* was not detectable at baseline, but it gradually increased, peaking at the 3rd hour of PTIVA. Subsequently, TNF-*α* gradually decreased to undetectable levels by the 7th hour. Although the decrease in TNF-*α* levels may have been promoted by the anti-inflammatory effects of IL-6 [32], TNF-*α* remained elevated for approximately 6 hours, increasing the possibility of organ injury [27]. Tumor necrosis factor alpha may contribute to the pathology of postoperative myocardial infarction, postischemic cardiac dysfunction, and heart failure [3]. Circulating TNF-*α* may interact with specific receptors on endothelial cells, inducing cell adhesion, apoptosis, endothelial permeability, and eventually lung injury [3]. It may also enhance neutrophil recruitment into the hepatic vascular beds, increasing the production of reactive oxygen species and promoting hepatic injury [33]. Furthermore, systemic inflammation and high levels of TNF-*α* can bias hematopoiesis toward myeloid-cell production, inhibit erythroid-precursor proliferation, shorten the erythrocyte lifespan, and inhibit the release of recycled iron from macrophages (mediated by IL-6), causing hypoferremia and anemia, which may increase complications and mortality in surgical and critically ill patients [34].

In addition, the increased inflammatory response and TNF-*α* levels may exacerbate brain injury, inhibit stimulation of neurogenesis, and cause neuroinflammation and blood-brain barrier disruption [3]. Also, TNF-*α* has a physiological role in synaptic transmission and plasticity in the healthy central nervous system, but increased TNF-*α* levels have an inhibitory effect on glutamate transporters, resulting in increased glutamate concentration and excessive toxicity [35]. The expression of mTNF-*α* (a precursor of TNF-*α*) in microglia cells increases during propofol anesthesia and may mediate propofol-induced neurotoxicity and cognitive impairment in surgical or critically ill patients [31]. Also, animal studies have shown that propofol anesthesia for five hours may cause neuronal and oligodendrocyte death in fetal and neonatal brain, while even subanesthetic doses of propofol can induce neuroapoptosis [36]. All these may have devastating effects on the developing human brain. Our study adds to the evidence showing that TNF-*α* may be implicated in PTIVA-related modulation of neuronal activity and could also interfere in physiological brain development. As there are no alternatives to general anesthetic drugs for some patients or children younger than 3 years of age, our findings may be important in translational research aiming at the elucidation of the role of PTIVA and TNF-*α* in neurotoxicity and brain injury.

An advantage of this study is the investigation of the dynamic behavior of IL-6, PCT, and TNF-*α* through serial measurements and at predefined time points. More studies investigating a variety of cytokines during PTIVA are needed to elucidate the associated immunomodulation. We acknowledge a few limitations to the study. First, this is an animal study and may not accurately reflect human inflammatory response to anesthetics. The time to reach the maximum levels of IL-6, PCT, and TNF-*α* during maintenance of anesthesia may vary among species. Second, the instrumentation, lung ventilation, use of other drugs, and prolonged immobility may have influenced our results, but these also take place during anesthesia in humans. Lung-protective ventilation has not been studied in Landrace-Large White swine and therefore, we used a validated model in order to simulate general anesthesia [10]. Of note, the recommended tidal volume in Landrace-Large White swine is 15 ml/kg [9], but we used a tidal volume of 10 ml/kg for minimizing the effects of mechanical ventilation on the inflammatory response. Also, the immunomodulatory properties of common *i.v.* anesthetic agents must be further studied in different types of general anesthesia. Another limitation is the problems associated with biomarker detection and quantification, including the application of methodologies and assays of high sensitivity and specificity, and the determination of optimal study end-points. In addition, the exact time period during which a potential biomarker may reveal useful information varies. Assessing the appropriate duration of an experiment for the reliable monitoring of plasma levels of inflammatory markers is particularly challenging. In our study, the 8-hour monitoring period may simulate the duration of anesthesia for major surgery or the early phases of anesthesia in the ICU. Another question raised by this study is whether an extension of the duration of the experimental procedure would be more informative, e.g., to simulate prolonged PTIVA in the ICU.

## 5. Conclusions

This is the first report of baseline values and kinetics of IL-6, PCT, and TNF-*α* in healthy Landrace-Large White swine anesthetized with PTIVA. Considering the lack of evidence in humans and swine, more studies are necessary to better understand the influence of PTIVA on the kinetics of these mediators.

## Figures and Tables

**Table 1:** Hemodynamic and metabolic parameters of the animals.

	0 h	1 h	2 h	3 h	4 h	5 h	6 h	7 h	8 h	*p* value^∗^
Heart rate	104.5 ± 13.4	115 ± 21.2	113.5 ± 42.3	130.3 ± 41.4	129.9 ± 24	107.8 ± 14.1	99.3 ± 11	112.6 ± 32	116.5 ± 28.4	0.041
SAP	100 ± 22.8	93.1 ± 25.6	106.3 ± 20.4	99.3 ± 14.5	103.5 ± 14.8	102.4 ± 11	110.5 ± 12.5	115.5 ± 23.7	115 ± 10.2	0.038
DAP	61.8 ± 18.2	58.9 ± 21.4	57.6 ± 18.4	60.9 ± 22.4	73.5 ± 14.4	62.6 ± 19.3	66.1 ± 23.6	75.1 ± 17.3	82.4 ± 8.3	0.036
MAP	74.5 ± 19.6	68.8 ± 24.7	73.8 ± 18.6	73.6 ± 19.6	83.5 ± 14.1	75.7 ± 16.2	80.8 ± 19.6	88.5 ± 19.1	93.2 ± 8.6	0.044
SpO_2_	94.8 ± 1	94.5 ± 1	94.7 ± 1.2	95.3 ± 1	95 ± 1	95 ± 1	95.1 ± 1	95 ± 1.3	95.3 ± 1	0.820
pH	7.41 ± 0.2	7.40 ± 0.2	7.40 ± 0.1	7.41 ± 0.2	7.40 ± 0.2	7.40 ± 0.2	7.41 ± 0.1	7.41 ± 0.2	7.40 ± 0.1	0.611
PaO_2_	92.9 ± 0.1	93 ± 0.5	92.5 ± 1	93.4 ± 0.5	92.5 ± 1	93 ± 1	92.3 ± 1	93 ± 0.8	93 ± 1	0.225
PaCO_2_	40.5 ± 0.8	39.8 ± 0.7	40.6 ± 0.8	40.3 ± 0.7	40.6 ± 0.7	40.3 ± 0.5	40.8 ± 1	40.4 ± 0.5	40.3 ± 0.7	0.228
PaO_2_/FiO_2_	442.4 ± 5	443 ± 2.6	440 ± 4.5	444 ± 3.4	440.5 ± 4.6	442.4 ± 4.2	440.5 ± 6	443 ± 3.7	443 ± 3.7	0.522
HCO_3_	27.8 ± 0.5	28 ± 0.7	28 ± 0.7	28.3 ± 0.6	28 ± 0.5	27.6 ± 0.7	28.3 ± 0.7	28 ± 0.5	28 ± 0.7	0.756
Base excess	4.5 ± 0.2	9.6 ± 1.4	9.8 ± 1.5	9.5 ± 1.3	4.7 ± 0.2	9.5 ± 1.4	9.5 ± 1.4	9.7 ± 1.4	9.2 ± 1.3	0.981
Lactate	1.8 ± 0.2	1.8 ± .02	1.6 ± 0.22	1.7 ± 0.3	1.6 ± 0.2	1.8 ± 0.3	1.9 ± 0.3	1.9 ± 0.3	1.8 ± 0.3	0.665

Values are expressed as median. ^∗^Friedman test for repeated measures. SAP: systolic arterial pressure; DAP: diastolic arterial pressure; MAP: mean arterial pressure; SpO_2_: saturation of peripheral oxygenation; PaO_2_: arterial partial pressure of oxygen; PaCO_2_: arterial partial pressure of carbon dioxide; FiO_2_: fraction of inspired oxygen.

**Table 2:** Complete blood count and serum chemistry analysis during the 8-hour period.

	0 h	1 h	2 h	3 h	4 h	5 h	6 h	7 h	8 h	*p* value^∗^
WBC	8600	11200	6600	6000	6200	8700	11600	12600	13100	0.757
PLT	335	327	308	311	286	268	234	289	276	0.393
NEUT	22.5	55	35.5	51	52.5	57	64	59	69	0.285
LYMPH	69	38	58	45	43.5	39	35	35	30	0.287
HB	9.1	9.2	9.6	9.8	9.1	8.9	8.4	8.9	8.7	0.940
HCT	29	30	30	31	28	30	27.5	28	27	0.775
ALT	33	34	30	33.5	31	30	33	31	31	0.970
AST	32	34	36	49	37	42	62	41	43	0.508
CREA	0.6	0.7	0.7	0.75	0.7	0.7	0.9	0.8	0.9	0.075
UREA	19	22	23	26	24	23	30	30	31	0.010
GGT	32	29	32	47.5	44	38	27	24	21	0.480
CK	1196	1341	1232	1099	972	547	1259	1016	1092	0.879

Values are expressed as median. ^∗^Friedman test for repeated measures. WBC: white blood cells; PLT: platelets; NEUT: neutrophils; LYMPH: lymphocytes; HB: hemoglobin; HCT: hematocrit; ALT: alanine aminotransferase; AST: aspartate aminotransferase; GGT: gamma-glutamyl transferase; CREA: creatinine; CK: creatine kinase.

**Table 3:** Variation of cytokines during the 8-hour period.

	0 h	1 h	2 h	3 h	4 h	5 h	6 h	7 h	8 h	*p* value^∗^
IL-6 (pg/ml)	18 (8-23)	65 (49-122)	89 (46-150)	44 (16-352)	36 (15-58)	2 (0.03-34)	17 (0.03-731)	34 (17-43)	26 (2-778)	<0.0001
Procalcitonin (ng/ml)	21 (18-24)	20 (18-22)	28 (17-68)	28 (25-105)	27 (24-268)	30.5 (21-32)	35 (22-35)	26 (24-28)	25 (20-41)	0.152
TNF-*α* (pg/ml)	ND	25 (22-27)	43 (42-45)	54 (51-55)	36 (34-37)	15 (14-16)	15 (13-16)	ND	ND	<0.001

Values are expressed as median (±SD). ^∗^Friedman test for repeated measures. TNF-*α*: tumor necrosis factor alpha; IL-6: interleukin-6; ND: nondetectable.

## Data Availability

Data from this study can be made available upon request through a collaborative process. Please contact thanoschalkias@yahoo.gr for additional information.
